# Patterns of engagement with the health care system and risk of subsequent hospitalization amongst patients with diabetes

**DOI:** 10.1186/1472-6963-13-399

**Published:** 2013-10-09

**Authors:** Paul E Ronksley, Pietro Ravani, Claudia Sanmartin, Hude Quan, Braden Manns, Marcello Tonelli, Brenda R Hemmelgarn

**Affiliations:** 1 Department of Community Health Sciences, Faculty of Medicine, University of Calgary, Calgary, Canada; 2 Department of Medicine, Faculty of Medicine, University of Calgary, Calgary, Canada; 3 Health Analysis Division, Statistics Canada, Ottawa, Ontario, Canada; 4 Department of Medicine, Faculty of Medicine, University of Alberta, Edmonton, Canada

**Keywords:** Diabetes, Administrative data, Hospitalization

## Abstract

**Background:**

Re-hospitalization is common among patients with diabetes, and may be related to aspects of health care use. We sought to determine the association between patterns of health care engagement and risk of subsequent hospitalization within one year of discharge for patients with diabetes.

**Methods:**

We identified adults with incident diabetes in Alberta, Canada, who had at least one hospitalization following their diabetes diagnosis between January 1, 2004 and March 31, 2011. We used Cox regression to estimate the association between factors related to health care engagement (prior emergency department use, primary care visits, and discharge disposition (i.e. whether the patient left against medical advice)) and the risk of subsequent all-cause hospitalization within one year.

**Results:**

Of the 33811 adults with diabetes and at least one hospitalization, 11095 (32.8%) experienced a subsequent all-cause hospitalization within a mean (standard deviation) follow-up time of 0.68 (0.3) years. Compared to patients with no emergency department visits, there was a 4 percent increased risk of a subsequent hospitalization for every emergency department visit occurring prior to the index hospitalization (adjusted Hazard Ratio [HR]: 1.04; 95% CI: 1.03–1.05). Limited and increased use of primary care was also associated with increased risk of a subsequent hospitalization. Compared to patients with 1–4 visits, patients with no visits to a primary care physician (adjusted HR: 1.11; 95% CI: 0.99–1.25) and those with 5–9 visits (adjusted HR: 1.06; 95% CI: 1.00–1.12) were more likely to experience a subsequent hospitalization. Finally, compared to patients discharged home, those leaving against medical advice were more likely to have a subsequent hospitalization (adjusted HR: 1.74; 95% CI: 1.50–2.02) and almost 3 times more likely to have a diabetes-specific subsequent event (adjusted HR: 2.86; 95% CI: 1.82–4.49).

**Conclusions:**

Patterns of health care use and the circumstances surrounding hospital discharge are associated with an increased risk of subsequent hospitalization among patients with diabetes. Whether these patterns are related to the health care systems ability to manage complex patients within a primary care setting, or to access to primary care services, remains to be determined.

## Background

Diabetes affects approximately one in ten adults in Canada [1,2] with treatment costs estimated to exceed $12 billion dollars per year [3]. A large component of costs are attributed to the direct costs of inpatient care, despite the fact that diabetes is a chronic condition generally amenable to outpatient treatment [4-6]. Patients with diabetes have an increased risk of hospitalization compared to those without diabetes [7-10]. Further, repeated hospitalizations are also common and, although observed in a smaller proportion of patients with diabetes, may represent a disproportionate burden on the health care system [11-15].

Since re-hospitalization is common and an important driver of morbidity and costs in diabetes [11-15], identifying which patients are at highest risk of subsequent hospitalization is relevant. Given the multiple factors which might contribute to re-hospitalization, the Andersen Behavioral Model for health care utilization can be used as a framework to identify the important patient and provider/system level factors [16]. Prior studies have identified patient level factors including demographic (age, sex, race, and socioeconomic status), clinical (comorbidity, diabetes duration), and behavioral factors (glycemic control, and adherence to medication) [11-15,17], though many of these studies were restricted to pediatric or elderly patients with diabetes limiting the generalizability of their findings. Furthermore, few studies have assessed aspects of patient care related to patterns of engagement with the health care system, including use of primary care, emergency rooms, and the circumstances around hospital discharge (including destination and whether people leave against medical advice).

Given the limitations in previous research and the burden that repeated hospitalization places on the health care system, we used population-based data to determine the association between patterns of health care engagement (health resource utilization and discharge disposition), and the risk of subsequent hospitalization among patients with diabetes.

## Methods

### Study population

We identified all adults (≥18 years) with incident diabetes and at least one hospitalization following diabetes diagnosis in the province of Alberta, Canada between January 1, 2004 and March 31, 2011. Eligible participants with diabetes were identified using an validated algorithm based on administrative data (two physician claims or one hospital discharge code for diabetes within a two-year period) [18]. The date on which the criteria for diabetes were met was defined as the participants’ diabetes diagnosis date. We identified the first (index) hospitalization, excluding pregnancy-related events, from the date of diabetes diagnosis until March 31, 2011. Subjects treated with dialysis or a kidney transplant prior to the index event (as determined from provincial renal program databases) were excluded [19], as they are a unique subgroup with high rates of hospitalization [20,21]. Participants that died during their index hospitalization were also excluded. This study cohort was derived from a previously described provincial laboratory repository [22].

### Measurement of health care engagement

We defined factors related to health care engagement from the administrative data files of the provincial health ministry (Alberta Health), including the number of emergency department visits and primary care physician visits in the year prior to the index hospitalization and the discharge disposition of the index event. We treated emergency department visits as a discrete continuous variable from 0 up to 1 visit per week (maximum of 52 events per year). Outpatient primary care physician visits were categorized into 0 visits, 1–4 visits, 5–9 visits, and ≥10 visits per year. Discharge disposition was categorized as: transfer to a palliative care setting, transfer to a long-term care facility, discharged home, discharged home with support services, or left against medical advice, as determined from the hospital database.

### Measurement of outcomes

We followed participants for a maximum of one year from discharge from their index hospitalization until a subsequent hospitalization, death, out-migration, or end of study follow up (March 31, 2011), whichever came first. The primary outcome was subsequent hospitalization, defined as an all-cause hospitalization (excluding pregnancy-related hospitalizations) within 1 year of discharge from the index (all-cause) hospitalization. A minimum of 1 day from the discharge date of the index hospitalization and admission date of the subsequent hospitalization was required to define a subsequent event. Secondary outcomes included time to subsequent hospitalization for cardiovascular (acute myocardial infarction [AMI], congestive heart failure [CHF], stroke) and diabetes-specific outcomes irrespective of the diagnosis from the index hospitalization. Cardiovascular outcomes were identified using validated administrative algorithms [23-25] and diabetes-specific hospitalizations were identified using pre-specified International Classification of Diseases, Tenth Revision (ICD-10) codes within the most responsible diagnosis field (Additional file 1: Table S1).

### Measurement of covariates

We identified covariates of interest based on the Andersen Behavioral Model [16]. Patient-level characteristics included age, sex, urban/rural status, First Nations Status, neighborhood median household income quintile, and diabetes duration. Comorbidities included hypertension, affective disorder, and conditions defined in the Charlson comorbidity index [26]. We identified hypertension from hospital discharge records and physician claims based on validated algorithms [27]. Affective disorder was defined as at least two physician claims or 1 hospitalization coding for affective disorder in a 3-year period prior to the index hospitalization. Additional comorbid conditions from the Charlson comorbidity index were identified using validated ICD-10 coding algorithms [28] and the presence of 1 or more diagnostic code in any position up to 3 years prior to the index hospitalization. Using provincial laboratory data sources, we determined whether a participant had at least one A1c measurement in the 6-month period prior to their index hospitalization. We also identified the most recent serum creatinine measurement in the same time period to estimate the kidney function (estimated glomerular filtration rate [eGFR]) using the CKD-EPI equation [29]. eGFR was categorized as ≥90, 60 to 89.9, 45 to 59.9, 30 to 44.9, 15 to 29.9, and <15 mL/min/1.73 m^2^. Characteristics related to the index hospitalization included length of stay, hospitalization type (emergent/urgent or elective), and the most responsible diagnosis of the index event (categorized based on ICD-10 chapters). Finally, we determined the rate of outpatient primary care physician visits in the year following discharge from the index hospitalization as a measure of post-discharge care.

### Statistical analysis

Participant characteristics were described using proportions, means (standard deviation (SD)), and medians (inter-quartile range (IQR)) where appropriate. We used Cox proportional hazards regression to study the association between patterns of engagement with the health care system, (including use of primary care, emergency rooms), and the circumstances around hospital discharge, and time to subsequent all-cause hospitalization. Initially, unadjusted hazard ratios (HRs) were calculated for all health resource use/discharge disposition variables of interest. We compared the hazard of subsequent hospitalization by the number of primary care physician visits in the year prior to their index hospitalization (0 visits, 5–9 visits, ≥10 visits per year) compared to those with 1–4 visits per year. The hazard of subsequent hospitalization by discharge disposition was also compared against those discharged home (reference group). Emergency department visits in the year prior to the index hospitalization was modeled as a continuous variable.

We developed multivariate models based on the identification of significant predictors of subsequent hospitalization. Using a stepwise model building approach, we added the following groups of variables separately into an adjusted model: socio-demographic variables, comorbidities, and factors related to the index hospitalization and post discharge care. For the neighborhood median household income quintile and level of kidney function (eGFR) variables, “missing” was included as a separate category due to the number of respondents with missing data for these variables. Finally, we used backwards elimination techniques to develop reduced models based on the presence or absence of effect modification and confounding by the specified predictors. Variables were retained based on their potential confounding effect (≥10% change of the exposure coefficients) or if they had a significant independent effect on outcomes. Our analysis was repeated for the outcomes of time to subsequent hospitalization for cardiovascular and diabetes-specific causes. The proportional hazard assumption was evaluated and satisfied for all bivariate and multivariable adjusted survival analyses. Model fit was also assessed graphically using standard methods.

We did two sensitivity analyses to assess the robustness of our study findings. First, to ensure that all patients had sufficient time to experience the outcome of interest, we limited our cohort to those with at least one year of follow-up from discharge of their index event until March 31, 2011. Second, we assessed the competing risk of death on the primary outcome according to methods of Fine and Gray [30]. For all statistical tests, P < 0.05 was considered statistically significant. Statistical analyses were done using STATA version 11.2 (http://www.stata.com). The Conjoint Health Research Ethics Board of the University of Calgary approved this study and granted waiver of patient consent.

## Results

### Cohort formation and characteristics

**Figure 1:**
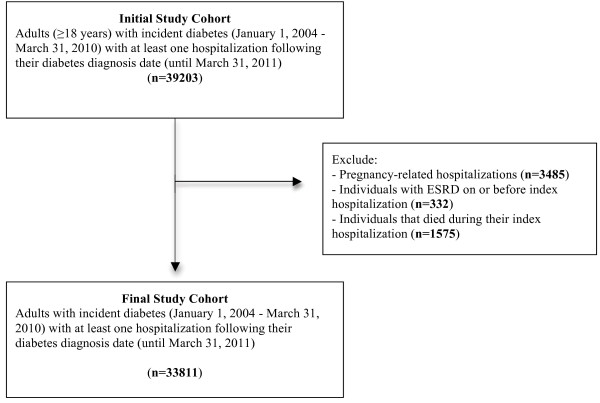
Flow diagram of cohort formation.

We identified 39203 subjects 18 years of age and older with incident diabetes and at least one hospitalization between January 1, 2004 and March 31, 2011. We excluded 5392 subjects (Figure 1), for a final study cohort of 33811. The mean age (SD) of the cohort was 63.3 (15.4) years and 53.4% were male (Table 1). Chronic obstructive pulmonary disease (COPD), hypertension, cancer, and CHF were the most common comorbidities. Table 2 describes the measures of health care use and characteristics of the index hospitalization. In the 1-year period prior to the index event, 3.4% and 44.1% of patients had no visits to an outpatient primary care physician or emergency department respectively. Injury/accident and diagnoses related to the circulatory system were the most common most responsible diagnoses for the index hospitalization. Approximately 80% of patients were discharged home from the index hospitalization with 1.1% of patients signing out against medical advice.

**Table 1:** Participant characteristics (n = 33811)

**Characteristics**	N (%)*
Age - years, Mean (SD)	63.3 (15.4)
Age Categories	
18-49	6509 (19.3)
50-64	11218 (33.2)
65-74	7639 (22.6)
75+	8445 (24.9)
Male	18051 (53.4)
Rural Residence	6541 (19.4)
First Nations Status	1662 (4.9)
Median Neighborhood Household Income	
1^st^ quintile (lowest)	8086 (23.9)
2^nd^ quintile	7316 (21.6)
3^rd^ quintile	6423 (19.0)
4^th^ quintile	5985 (17.7)
5^th^ quintile (highest)	5200 (15.4)
Missing	801 (2.4)
Diabetes Duration - years, Median (IQR)	1.2 (0.4–2.6)
Charlson Comorbidities	
Cancer	6299 (18.6)
Cerebrovascular Disease	4127 (12.2)
Congestive Heart Failure (CHF)	5435 (16.1)
Chronic Obstructive Pulmonary Disease (COPD)	10269 (30.4)
Dementia	2275 (6.7)
HIV/AIDS	50 (0.2)
Metastatic Solid Tumor	1496 (4.4)
Myocardial Infarction	5098 (15.1)
Mild Liver Disease	1188 (3.5)
Moderate/Severe Liver Disease	408 (1.2)
Para/Hemiplegia	803 (2.4)
Peptic Ulcer Disease	1611 (4.8)
Peripheral Vascular Disease	2676 (7.9)
Renal Disease	2714 (8.0)
Rheumatologic Disease	1225 (3.6)
Hypertension	7095 (21.0)
Affective Disorder	3340 (9.9)
At least 1 A1c measurement in 6 month period prior to index hospitalization	16698 (49.4)
eGFR Category (mL/min/1.73 m^2^)	
≥90	7220 (21.4)
89-60	10391 (30.7)
59-45	3210 (9.5)
44-30	1722 (5.1)
29-15	625 (1.8)
<15	132 (0.4)
No measurement in 6 month period prior to index hospitalization	10511 (31.1)

*All values expressed as proportions unless otherwise specified.

**Table 2:** Patterns of health care engagement and characteristics of the index hospitalization

**Characteristics**	N (%)*
Number of Primary Care Physician Visits†	
0	1133 (3.4)
1-4	7655 (22.6)
5-9	12487 (36.9)
10+	12536 (37.1)
Rate of Primary Care Physician Visits Post Discharge, (visits/year) median (IQR)	9.2 (5.0-18.0)
Number of Emergency Department Visits†	
0	14915 (44.1)
1	8161 (24.1)
2	4216 (12.5)
3+	6519 (19.3)
Length of stay during index hospitalization, days, median (IQR)	5 (3–10)
<5	14730 (43.6)
5-9	10044 (29.7)
10+	9037 (26.7)
Hospitalization type for index hospitalization	
Elective	10034 (29.7)
Emergent/Urgent	23777 (70.3)
Most Responsible Diagnosis of index hospitalization	
Cancer	2999 (8.9)
Mental Health	1627 (4.8)
Circulatory	5565 (16.4)
Respiratory	2559 (7.6)
Digestive	4154 (12.3)
Musculoskeletal	3129 (9.2)
Urinary	2662 (7.9)
Injury/Accident	6152 (18.2)
Other	4964 (14.7)
Discharge disposition of index hospitalization	
Transferred to long-term care facility	1415 (4.2)
Transferred to palliative/hospice	129 (0.4)
Discharged to home setting with support services	4754 (14.1)
Discharged home	27123 (80.2)
Signed out against medical advice	390 (1.1)

*All values expressed as proportions unless otherwise specified.

†In 1-year period prior to index hospitalization.

### Association between patterns of engagement with the health care system and all-cause subsequent hospitalization

**Figure 2:**
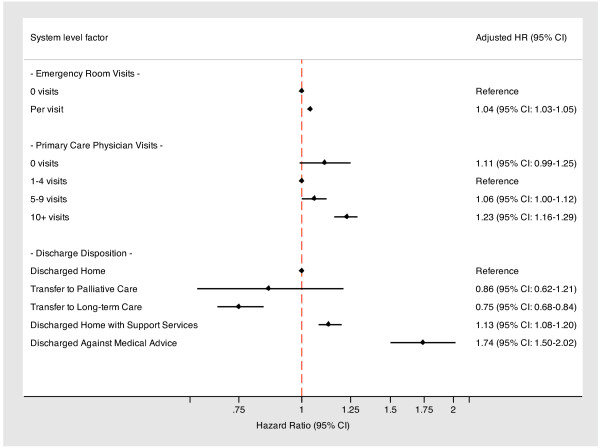
**Association between patterns of health care engagement and subsequent *all-cause *hospitalization.**

The mean (SD) follow-up time for subjects was 0.68 (0.3) years. During this study period, 11095 patients (32.8%) with diabetes experienced a subsequent all-cause hospitalization, 1033 (9.3%) died after their index hospitalization, and 355 (3.2%) out-migrated from the province. After adjusting for patient-level characteristics and factors related to the index hospitalization, we found that emergency department visits, primary care physician visits, and discharge disposition were all associated with an increased risk of subsequent hospitalization (Figure 2). Compared to subjects with no emergency department visits in the 1-year period prior to the index hospitalization, there was a 4% increased risk of a subsequent hospitalization for every additional visit (adjusted HR: 1.04; 95% confidence interval [CI]: 1.03–1.05). Though not statistically significant, patients with no visits to a primary care physician appeared more likely to have a repeat hospitalization compared to those with 1–4 visits (adjusted HR: 1.11; 95% CI: 0.99–1.25), while patients with five or more visits were significantly more likely to experience a subsequent hospitalization (5–9 visits; adjusted HR: 1.06; 95% CI: 1.00–1.12; 10+ visits; adjusted HR: 1.23; 95% CI: 1.16–1.29). Finally, compared to patients discharged home, those discharged home with support services were more likely to have a subsequent all-cause hospitalization. This risk was almost two-fold higher for patients that left against medical advice (adjusted HR: 1.74; 95% CI: 1.50–2.02) (Table 3).

**Table 3:** **Patterns of health care engagement associated with subsequent *all-cause *hospitalization within 1 year of discharge from an index hospitalization among patients with diabetes**

	**Unadjusted model**	Multivariate adjusted model*
	**HR (95% CI)**	**HR (95% CI)**
# of emergency department visits in the 1-year period		
Prior to the index hospitalization		
0	Reference	Reference
Per visit	1.05 (1.04–1.06)	1.04 (1.03–1.05)
# of primary care physician visits in the 1-year period		
Prior to the index hospitalization		
0	1.26 (1.13–1.41)	1.11 (0.99–1.25)
1-4	Reference	Reference
5-9	1.08 (1.02–1.14)	1.06 (1.00–1.12)
10+	1.48 (1.41–1.56)	1.23 (1.16–1.29)
Discharge Disposition of index hospitalization		
Discharged Home	Reference	Reference
Transfer to Palliative Care	1.46 (1.04–2.03)	0.86 (0.62–1.21)
Transfer to Long-term Care	1.29 (1.17–1.41)	0.75 (0.68–0.84)
Discharged Home with Support Services	1.55 (1.48–1.63)	1.13 (1.08–1.20)
Left Against Medical Advice	1.85 (1.60–2.13)	1.74 (1.50–2.02)

*Adjustment for patient level factors (Age, sex, diabetes duration, neighborhood income quintile, urban/rural status, First Nations status, A1c measurement in past 6 months (Y/N), eGFR category prior to index hospitalization, hypertension, affective disorder, Charlson comorbidities (cancer, congestive heart failure, COPD, dementia, metastatic solid tumor, myocardial infarction, mild liver disease, moderate/severe liver disease, paraplegia/hemiplegia, peptic ulcer disease, peripheral vascular disease, renal disease, rheumatic disease) and factors related to index hospitalization (most responsible diagnosis and length of stay) and health resource use post discharge.

### Association between patterns of engagement with the health care system and cause-specific subsequent hospitalization

A total of 867 cardiovascular-specific and 409 diabetes-specific subsequent hospitalizations were identified in the 1-year follow-up period. Similarly, each additional emergency department visit in the year prior to the index event was associated with an increased risk of cardiovascular-specific and diabetes-specific subsequent hospitalization compared to patients with no emergency department visits (Table 4). There was no association between primary care physician visits and cause-specific subsequent hospitalization. Patients that left against medical advice were more than twice as likely to have a subsequent cardiovascular-specific hospitalization (adjusted HR: 2.11; 95% CI: 1.13–3.97) and almost 3 times more likely to have a diabetes-specific repeat event (adjusted HR: 2.86; 95% CI: 1.82–4.49).

**Table 4:** **Patterns of health care engagement associated with subsequent *cardiovascular and diabetes-specific *hospitalization within 1 year of discharge from an index hospitalization among patients with diabetes**

	**Cardiovascular-specific**		**Diabetes-specific**	
	**Unadjusted model**	Adjusted model*	**Unadjusted model**	**Adjusted model†**
	**HR (95% CI)**	**HR (95% CI)**	**HR (95% CI)**	**HR (95% CI)**
# of emergency department visits in the 1-year				
Period prior to the index hospitalization				
0	Reference	Reference	Reference	Reference
Per visit	1.05 (1.03–1.06)	1.03 (1.01–1.05)	1.05 (1.03–1.07)	1.03 (1.01–1.05)
# of primary care physician visits in the 1-year				
Period prior to the index hospitalization				
0	1.11 (0.73–1.68)	0.95 (0.63–1.44)	1.71 (1.16–2.52)	1.08 (0.73–1.60)
1-4	Reference	Reference	Reference	Reference
5-9	1.05 (0.86–1.27)	0.97 (0.80–1.18)	0.54 (0.42–0.69)	0.78 (0.61–1.02)
10+	1.54 (1.29–1.85)	1.10 (0.91–1.33)	0.63 (0.50–0.81)	0.96 (0.74–1.26)
Discharge Disposition of index hospitalization				
Discharged Home	Reference	Reference	Reference	Reference
Transfer to Palliative Care	--	--	1.11 (0.16–7.88)	1.24 (0.17–9.00)
Transfer to Long-term Care	2.14 (1.62–2.82)	0.78 (0.58–1.05)	0.49 (0.24–1.05)	0.63 (0.29–1.37)
Discharged Home with Support Services	2.34 (2.00–2.73)	1.26 (1.07–1.50)	1.14 (0.86–1.51)	1.32 (0.98–1.81)
Left Against Medical Advice	1.38 (0.74–2.58)	2.11 (1.13–3.97)	5.54 (3.60–8.54)	2.86 (1.82–4.49)

*Adjustment for patient level factors (Age, sex, urban/rural status, A1c measurement in past 6 months (Y/N), eGFR category prior to index hospitalization, hypertension, Charlson comorbidities (cerebrovascular disease, congestive heart failure, myocardial infarction, renal disease), factors related to index hospitalization (most responsible diagnosis and length of stay) and health resource use post discharge.

†Adjustment for patient level factors (Age, sex, urban/rural status, A1c measurement in past 6 months (Y/N), eGFR category prior to index hospitalization, hypertension, affective disorder, Charlson comorbidities (cerebrovascular disease, congestive heart failure, myocardial infarction, peripheral vascular disease), factors related to index hospitalization (most responsible diagnosis and length of stay) and health resource use post discharge.

### Sensitivity analyses

Sensitivity analyses excluding patients with less than one year of follow-up (n = 6138) did not change the associations between the exposures of interest and the risk of subsequent all-cause or cause-specific hospitalization (Additional file 2: Table S2). Treating death after discharge from the index hospitalization as a competing risk had a minor impact on the observed associations between our exposures of interest and risk of subsequent all-cause hospitalization (Additional file 3: Table S3). In a competing risks regression model, subjects with no primary care physician visits in the year prior to the index event were significantly more likely to have the outcome of interest. In addition, subjects discharged to palliative care or long-term care settings were significantly less likely. All remaining point estimates were similar to those observed in our multivariate Cox proportional hazards model.

## Discussion

In this large, population-based cohort of adults with diabetes and at least one hospitalization, we found that certain patterns of engagement with the health care system prior to the initial hospitalization, specifically higher use of the emergency department, and limited or increased use of primary care, were associated with an increased risk of subsequent hospitalization. Moreover, patients discharged against medical advice were more likely to be re-hospitalized. Given the financial burden that in-patient care places on the health care system, the ability to identify patients at highest risk of subsequent hospitalization is not only hypothesis generating, but may help healthcare providers target resources to high-risk patients.

Our results add to those from previous studies. Smith et al. found that the number of emergency department visits in the 6-month period prior to hospitalization was a significant predictor of 90-day repeat hospitalization among patients with chronic disease, some of whom had diabetes [31]. Our results extend this finding to a large cohort of patients with diabetes, and demonstrate similar risk associated with both all-cause and cause-specific subsequent hospitalization. The higher risk of re-hospitalization among patients with diabetes who have a higher use of the emergency department visits may reflect a sicker patient population with multi-morbidity [5,32,33], though we noted the same association after controlling for measured comorbidity. It is also possible it reflects patients with more severe diabetes, or those who are prone to hospitalizations related to hypo or hyperglycemia; hospitalizations that might be prevented by appropriate access and use of primary care services [34,35]. Regardless of the cause, higher use of the emergency department does appear to identify a group of patients at higher risk of re-hospitalization.

We also observed a relationship between the number of primary care physician visits and risk of subsequent all-cause hospitalization. In various chronic disease populations, increased primary care accessibility and use has been associated with decreased risk of hospitalization, especially for ambulatory care sensitive conditions [36,37]. In diabetes populations specifically, multiple physician visits have been shown to be associated with risk of first hospitalization [38]. Our results suggest that a higher number of primary care physician visits are also associated with a greater risk of subsequent hospitalization, possibly because those with multiple visits are sicker patients who require more complex care. Though our results suggest that the majority of patients have adequate access to primary care services, as observed by the rate of use both before and after hospital discharge, it may be that our current model of providing care is not adequate for management of complex patients in a primary care setting. A multidisciplinary approach to chronic disease care has been proposed and shown to reduce the risk of hospitalization specifically in patients with diabetes [39]. In addition, we found a potential increased risk of subsequent all-cause hospitalization in patients with no general physician visits prior to their index event. While limited to a small proportion of the study population and only significant in our sensitivity analysis, these results support previous literature showing that limited access is associated with increased hospitalization risk in chronic disease populations [40-42]. Future work is required to identify characteristic of these patients at high risk and determine whether the absence of health care use in the period prior to hospitalization represents limited access or health behaviors in which a patient chooses not to seek care.

A unique finding of our study was the association between discharge disposition, whether a patient left against medical advice, and risk of repeated hospitalization. Specifically, we found that patients discharged to palliative or long-term care were less likely to have a subsequent hospitalization (possibly given the competing risk of death) whereas those who left against medical advice were significantly more likely. In a cohort of elderly patients with diabetes identified within the California State Inpatient Dataset, Kim et al. found that a discharge disposition other than home was associated with an increased risk for an unscheduled subsequent hospitalization (OR: 1.28; 95% CI: 1.24–1.32) [14]. However, their dichotomous analysis could not determine how different transitions of care place patients with diabetes at different risk for subsequent hospitalization. Patients with diabetes who leave against medical advice represent a high-risk group worthy of future study to better understand the circumstances surrounding the discharge against medical advice.

Our study should be interpreted in light of its limitations. First, there are a number of factors that place patients at increased risk of subsequent hospitalization, including severity of disease, and thus, the possibility of residual confounding exists given our administrative data sources. However, we were able to adjust for a number of patient and clinical characteristics, including laboratory tests, which represent proxy measures of disease severity. Second, we were unable to determine whether a patient had a regular primary care physician, or the level of coordination available during the transition from the hospital to community care. Continuity of care and physician accessibility has been associated with improved outcomes, particularly in diabetes [40-42]. Our inability to adjust for these factors may also confound the observed associations. Finally, we assessed all-cause and cause-specific subsequent hospitalizations irrespective of the index hospitalization diagnosis, which makes the interpretation of these associations less clear. However, patients with diabetes often suffer from various micro and macro-vascular complications, and studies have shown that patients with diabetes are often hospitalized due to one or more of these complications [43]. Further, any hospitalization (regardless of type) represents a burden on the health care system. For these reasons, we elected to consider all hospitalizations that occurred among the study population.

Despite these limitations, our study has a number of strengths. We utilized population-based data within a single province of Canada, which provides a unique opportunity to comprehensively assess the issue of subsequent hospitalization in patients with diabetes. We also grounded this work in a recognized framework for the study of health care utilization (the Andersen Behavioral Model), and our results highlight the need for researchers and clinicians to consider health resource use and discharge disposition in context of the known patient-level and clinical factors that place diabetes patients at risk for repeated hospitalization. Unlike many patient and clinical characteristics, these associations observed could be considered modifiable and represent areas that require further exploration.

## Conclusions

In summary, we found certain patterns of engagement with the health care system are associated with an increased risk of subsequent hospitalization among patients with diabetes, including increased frequency of emergency department visits, limited and increased use of primary care visits, as well as leaving the hospital against medical advice. We acknowledge that repeated hospitalization is a complex topic that requires an understanding of the multiple patient, provider and system level factors that influence it. Though subsequent hospitalization may represent progression in the natural history of the patient’s underlying disease, or the consequences of poor coordination of care following discharge, our findings should be considered hypothesis-generating and represent an important step in the development of strategies to identify and intervene on patients at high risk of re-hospitalization. Our results also highlight the need to consider the patterns of health care engagement when studying re-hospitalization among patients with diabetes. Inclusion of these factors may ultimately improve predictive accuracy of this outcome in future studies.

## Abbreviations

AMI: Acute myocardial infarction; CHF: Congestive heart failure; CI: Confidence interval; COPD: Chronic obstructive pulmonary disease; eGFR: Estimated glomerular filtration rate; ICD-10: International classification of diseases, Tenth Revision; IQR: Inter-quartile range; HR: Hazard ratio; SD: Standard deviation.

## Competing interests

The authors declare that they have no competing interests.

## Authors’ contributions

PR was involved in the conception and design of the study. He was also responsible for drafting the manuscript, conducting the analysis, and interpreting the data. CS and PR contributed to conception and design and to interpretation of data, as well as providing intellectual content. HQ, MT, and BM contributed to the conception and design of the study and provided interpretation and intellectual content to subsequent drafts of the manuscript. BH also contributed to the study conception and design, data interpretation, and manuscript revisions. All authors read and approved the final draft. BH is the study guarantor.

## Pre-publication history

The pre-publication history for this paper can be accessed here:

http://www.biomedcentral.com/1472-6963/13/399/prepub

## Supplementary Material

Additional file 1: Table S1 ICD-10 codes for identification of cause-specific subsequent hospitalizations. Click here for file

Additional file 2: Table S2 Sensitivity analysis: Patterns of health care engagement associated with subsequent *all-cause, cardiovascular and diabetes-specific* hospitalization among patients with diabetes with at least 1 year of follow-up time. Click here for file

Additional file 3: Table S3 Sensitivity analysis using competing risk regression for the association between patterns of health care engagement and subsequent *all-cause* hospitalization. Click here for file
