# Supplementary material for: Patterns of engagement with the health care system and risk of subsequent hospitalization amongst patients with diabetes
Source: BMC Health Serv Res. 2013 Oct 9;13:399. doi: 10.1186/1472-6963-13-399 (PMC3851786; doi:10.1186/1472-6963-13-399)
Supplement: Additional file 1: Table S1 — ICD-10 codes for identification of cause-specific subsequent hospitalizations. [file 1472-6963-13-399-S1.docx]

Additional file 1. ICD-10 codes for identification of cause-specific subsequent hospitalizations

| Cardiovascular | ICD-10 Codes |
| --- | --- |
| Acute Myocardial Infarction | I21.x, I22.x, I25.2 |
| Congestive Heart Failure | I09.9, I11.0, I13.0, I13.2, I25.5, I42.0, I42.5-42.9, I43.x, I50.x, P29.0 |
| Stroke | H34.1, I60.x, I61.x, I63.x, I64.x, I67.7, G08, G45.x  (excluding G45.4) |
| Diabetes | ICD-10 Codes |
| Diabetes | E10.x – E14.x |
