# Supplementary material for: Patterns of engagement with the health care system and risk of subsequent hospitalization amongst patients with diabetes
Source: BMC Health Serv Res. 2013 Oct 9;13:399. doi: 10.1186/1472-6963-13-399 (PMC3851786; doi:10.1186/1472-6963-13-399)
Supplement: Additional file 2: Table S2 — Sensitivity analysis: Patterns of health care engagement associated with subsequent all-cause, cardiovascular and diabetes-specific hospitalization among patients with diabetes with at least 1 year of follow-up time. [file 1472-6963-13-399-S2.docx]

Additional file 2. Sensitivity analysis: Patterns of health care engagement associated with subsequent *all-cause,* *cardiovascular and diabetes-specific* hospitalization among patients with diabetes with at least 1 year of follow-up time

|  | All-cause | Cardiovascular-specific | Diabetes-specific |
| --- | --- | --- | --- |
|  | Adjusted Model*  HR (95% CI) | Adjusted Model†  HR (95% CI) | Adjusted Model††  HR (95% CI) |
| # of emergency department visits in the 1-year period prior to the index hospitalization  0  Per visit | Reference  1.04 (1.03–1.05) | Reference  1.03 (1.02–1.05) | Reference  1.04 (1.01–1.06) |
| # of primary care physician visits in the 1-year period prior to the index hospitalization  0  1-4  5-9  10+ | 1.13 (1.00–1.27)  Reference  1.06 (1.00–1.13)  1.23 (1.16–1.30) | 1.01 (0.66–1.54)  Reference  0.96 (0.78–1.18)  1.04 (0.85–1.28) | 1.10 (0.73–1.67)  Reference  0.80 (0.61–1.05)  0.92 (0.69–1.22) |
| Discharge Disposition of index hospitalization  Discharged Home  Transfer to Palliative Care  Transfer to Long-term Care  Discharged Home with Support Services  Left Against Medical Advice | Reference  0.96 (0.68–1.35)  0.76 (0.68–0.85)  1.13 (1.07–1.20)  1.62 (1.39–1.90) | Reference  --  0.68 (0.49–0.94)  1.19 (0.99–1.43)  2.13 (1.09–4.13) | Reference  1.45 (0.20–10.50)  0.52 (0.21–1.31)  1.42 (1.03–1.97)  2.77 (1.70–4.53) |

*All-cause model - Adjustment for patient level factors (Age, sex, diabetes duration, neighborhood income quintile, urban/rural status, First Nations status, A1c measurement in past 6 months (Y/N), eGFR category prior to index hospitalization, hypertension, affective disorder, Charlson comorbidities (cancer, congestive heart failure, COPD, dementia, metastatic solid tumor, myocardial infarction, mild liver disease, moderate/severe liver disease, paraplegia/hemiplegia, peptic ulcer disease, peripheral vascular disease, renal disease, rheumatic disease), factors related to index hospitalization (most responsible diagnosis and length of stay) and health resource use post discharge.

† Cardiovascular-specific model - Adjustment for patient level factors (Age, sex, urban/rural status, A1c measurement in past 6 months (Y/N), eGFR category prior to index hospitalization, hypertension, Charlson comorbidities (cerebrovascular disease, congestive heart failure, myocardial infarction, renal disease), factors related to index hospitalization (most responsible diagnosis and length of stay) and health resource use post discharge.

††Diabetes-specific model - Adjustment for patient level factors (Age, sex, urban/rural status, A1c measurement in past 6 months (Y/N), eGFR category prior to index hospitalization, hypertension, affective disorder, Charlson comorbidities (cerebrovascular disease, congestive heart failure, myocardial infarction, peripheral vascular disease), factors related to index hospitalization (most responsible diagnosis and length of stay) and health resource use post discharge.
