# Supplementary material for: Patterns of engagement with the health care system and risk of subsequent hospitalization amongst patients with diabetes
Source: BMC Health Serv Res. 2013 Oct 9;13:399. doi: 10.1186/1472-6963-13-399 (PMC3851786; doi:10.1186/1472-6963-13-399)
Supplement: Additional file 3: Table S3 — Sensitivity analysis using competing risk regression for the association between patterns of health care engagement and subsequent all-cause hospitalization. [file 1472-6963-13-399-S3.docx]

Additional file 3. Sensitivity analysis using competing risk regression for the association between patterns of health care engagement and subsequent *all-cause* hospitalization

|  | Adjusted Model  (Cox Proportional Hazards Model)  Hazard Ratio (95% CI) | Adjusted Model  (Fine and Gray Competing Risk Model)  Sub-hazard Ratio (95% CI) |
| --- | --- | --- |
| # of emergency department visits in the 1-year period prior to the index hospitalization  0  Per visit | Reference  1.04 (1.03–1.05) | Reference  1.03 (1.03–1.04) |
| # of primary care physician visits in the 1-year period prior to the index hospitalization  0  1-4  5-9  10+ | 1.11 (0.99–1.25)  Reference  1.06 (1.00–1.12)  1.23 (1.16–1.29) | 1.14 (1.02–1.27)  Reference  1.03 (0.98–1.09)  1.13 (1.06–1.19) |
| Discharge Disposition of index hospitalization  Discharged Home  Transfer to Palliative Care  Transfer to Long-term Care  Discharged Home with Support Services  Left Against Medical Advice | Reference  0.86 (0.62–1.21)  0.75 (0.68–0.84)  1.13 (1.08–1.20)  1.74 (1.50–2.02) | Reference  0.12 (0.07–0.20)  0.42 (0.36–0.48)  1.07 (1.02–1.14)  1.56 (1.34–1.83) |
